# Clonal Glial Response in a Multiple Sclerosis Mouse Model

**DOI:** 10.3389/fncel.2018.00375

**Published:** 2018-10-23

**Authors:** Ana Bribian, Fernando Pérez-Cerdá, Carlos Matute, Laura López-Mascaraque

**Affiliations:** ^1^Departamento de Neurobiología Molecular, Celular y del Desarrollo, Instituto Cajal-CSIC, Madrid, Spain; ^2^Centro de Investigación Biomédica en Red de Enfermedades Neurodegenerativas (CIBERNED), Leioa, Spain; ^3^Achucarro Basque Center for Neuroscience, Leioa, Spain; ^4^Departamento de Neurociencias, Universidad del País Vasco (UPV)/EHU, Leioa, Spain

**Keywords:** progenitor, EAE, cortex, lesion, StarTrack, astrocyte, reactive

## Abstract

Multiple sclerosis (MS) is an autoimmune disease causing central nervous system (CNS) demyelination and axonal injury. In the last years the importance of astrocytes in MS is rapidly increasing, recognizing astrocytes as highly active players in MS pathogenesis. Usually the role assigned to astrocytes in MS lesions has been the formation of the glial scar, but now their implication during lesion formation and the immune response increasingly recognized. Since astrocytes are a heterogeneous cell population with diverse roles in the CNS, the aim of this study was to analyze the putative clonal response of astrocytes in a demyelinating scenario. To undertake this aim, we used the induced experimental autoimmune encephalomyelitis (EAE) as a murine model for MS in previously electroporated mice with *in vivo* multicolor lineage tracing system, the StarTrack methodology. Our data revealed a variety of morphological changes that were different among distinct clones. In many cases, cells of the same clone responded equally to the injury, while in other cases clonally-related cells responded differently to the injury. Therefore, whereas some clones exhibited a strong morphological alteration, other clones located at similar distances to the lesion were apparently unresponsive. Thus, at present there is no compelling evidences that clonal relationship influences the position or function of astrocytes in the EAE model. Further, the coexistence of different astroglial clonal responses to the bran injury reveals the significance of development to determine the astrocyte features that respond to brain injuries.

## Introduction

Multiple sclerosis (MS) is a chronic, disabling autoimmune and neurological disorder targeting the white and gray matter of the central nervous system (CNS; Lassmann, 2012; Prins et al., 2015; van Munster et al., 2015). The loss of myelin and oligodendrocytes, axonal loss and glial scar formation in adult brain are the hallmarks for MS. In addition, activated microglia and astrocytes are also involved in the pathogenesis of MS, proliferating during MS progression (Correale, 2014; Correale and Farez, 2015; Ponath et al., 2018).

Current discoveries on the function of astrocytes in the brain in physiological and pathological conditions further highlighted the need to comprehensively evaluate the roles of these cells in all stages of MS (Brosnan and Raine, 2013; Ponath et al., 2018). Apart from the formation of the glial scar, astrocytes have other significant roles in pathogenesis, including a significant implication in the CNS innate immune response: they are a source of cytotoxic factors and provide support for oligodendrocytes and neurons (Brosnan and Raine, 2013).

Remarkably, reactive astrocytes suffer changes in both phenotype and gene expression in response to the distance to the injury, denoting a high degree of heterogeneity (Mathewson and Berry, 1985; Eddleston and Mucke, 1993; Ridet et al., 1997; Sofroniew, 2009). However, the heterogeneity in astrocytes is not exclusive to reactive cells. In fact, the concept of astrocyte heterogeneity emerged in the late nineteenth century based on morphological differences (Andrizen, 1893). During the last two decades, the development of new immunocytochemical, molecular and genetic techniques allowed researchers to identify differences between astrocytes originated/located in the same or different CNS regions (García-Marqués et al., 2010; Horii-Hayashi et al., 2010; Matyash and Kettenmann, 2010; García-Marqués and López-Mascaraque, 2013, 2017; Tabata, 2015; Bribián et al., 2016). In particular, we developed a multi-color lineage tracing system for astrocytes, the Star Track technology, based on the genomic incorporation of 12 plasmids encoding different fluorescent reporters driven by the GFAP promoter along with the transposase plasmid (García-Marqués and López-Mascaraque, 2013). Star Track provided *in vivo* evidence of the relationship between heterogeneity and lineage in astrocytes (García-Marqués and López-Mascaraque, 2013) as well as how groups of astrocyte clones respond differentially to cortical injury (Martín-López et al., 2013). Taking advantage of this technology, we analyzed the morphological response of clonally-related astrocytes to demyelinating lesions in murine myelin oligodendrocyte glycoprotein (MOG)-induced experimental autoimmune encephalomyelitis (EAE), the most commonly used animal model that resembles immunopathological and neurobiological aspects of MS. The positional identity of astrocyte clones in this brain pathology represents a new approach to unraveling some unknown aspects of pathogenesis of MS. Our data show that most astrocytes are similarly distributed whether or not they share a clonal relationship, while a large diversity of morphological changes were different among different clones.

## Materials and Methods

### Animals

Pregnant C57 mice from Janvier Labs were housed at the Universidad del País Vasco (UPV)-EHU animal facilities in standard cages, maintained under 12 h controlled light-dark cycles with food and water available *ad libitum*. This study was carried out in accordance with the recommendations of the ethical regulations on the use and welfare of experimental animals of the European Union (2010/63/EU) and the Spanish Ministry of Agriculture (RD 1201/2005 and L 32/2007). The protocol was approved by the Bioethical Committee at the UPV-EHU.

We analyzed cortical clones in two groups of mice: (1) nine adult StarTrack-electroporated mice (three sham and six EAE-induced mice); and (2) six adult StarTrack-electroporated animals (two sham and four EAE-induced mice). Sham animals were electroporated, with the exception of the fluid pulse.

### StarTrack Mixture

Clonal analysis was performed with the StarTrack approach (García-Marqués and López-Mascaraque, 2013, 2017). Briefly, StarTrack is based on the genomic incorporation of 12 plasmids encoding six different fluorescent reporter proteins, localized cytoplasm or nucleus, under the GFAP promoter. To allow the genomic integration of these constructs, each plasmid incorporates inverted terminal repeat sequences recognized by the piggyBac transposase plasmid. This results in a unique color combination code detecting glial clones derived from single progenitors. This will allow following cell dispersion of the progeny from embryonic mice single progenitor cells and generated an inheritable mark in astrocyte lineages.

### *In utero* Electroporation

Pregnant mice, at embryonic day 14 (E14), were deeply anesthetized by isofluorane inhalation (IsovaH vet, Centauro, 2 ml/L) and maintained at 37°C. After uterine horns were exposed, embryos were visualized by trans-illumination using an optical fiber. Using a glass micropipette, the plasmid mixture (2 ml, 2–5 mg DNA/ml containing 0.1% fast green) was injected into lateral ventricles (LV). Next, by using tweezer-type electrodes, one or two trains of five square pulses (35 V; 50 ms followed by 950 ms intervals) were delivered on each embryo. Uterine horns were placed back into abdominal cavity.

Finally, both abdominal and skin incisions, were sealed with absorbable polyglycolic acid (Surgicryl, Hünningen, BE) and silk (3/0 Lorca-Marin, Murcia, ES) sutures, respectively. Skin was cleaned with povidone-iodine and pregnant mice received a subcutaneous injection of both 5 mg/kg of the antibiotic enrofloxacin (Baytril; Bayer, Kiel, Germany) and 300 mg/kg of the anti-inflammatory/analgesic meloxicam (Metacam; Boehringer Ingelheim). Injected embryos were allowed to develop normally until they were born.

### EAE Induction and Treatment

EAE was induced in electroporated C57BL/6 mice by immunization with the immunodominant epitope of MOG (MOG 35–55). Chronic, relapsing EAE was induced successfully induced in male and female animals as previously described (Matute et al., 2007; Pampliega et al., 2011). Although gender differences in susceptibility have been described in mice (Voskuhl and Palaszynski, 2001; Teuscher et al., 2006), we used both males and females in this and previous studies without observing any significant gender difference in the symptoms (Matute et al., 2007; Pampliega et al., 2011; Saab et al., 2016), to minimize the use of animals to comply with institutional regulations. Briefly, C57BL/6 mice, 8-week-old, were immunized with 300 μg of MOG (35–55) (200 μg; Sigma Aldrich) in incomplete Freund’s adjuvant supplemented with 8 mg/ml *Mycobacterium tuberculosis* H37Ra. Pertussis toxin (500 ng; Sigma) was injected on the day of immunization and again 2 days later.

Motor symptoms were recorded daily and scored as follows from 0 to 8: 0, no detectable changes in muscle tone and motor behavior; 1, flaccid tail; 2, paralyzed tail; 3, impairment or loss of muscle tone in hindlimbs; 4, hindlimb hemiparalysis; 5, complete hindlimb paralysis; 6, complete hindlimb paralysis and loss of muscle tone in forelimbs; 7, tetraplegia; and 8, moribund. Later, mice were fixed by perfusion with 4% paraformaldehyde in phosphate buffer, post-fixed in the same fixation solution, and stored in phosphate-buffered saline (PBS) plus azide at 4°C.

### Histology and Immunohistochemistry

Brains were removed and vibratome sectioned in the coronal plane at 50 μm. After several washes in PBS-0.1% Triton (PBST), the sections were pre-incubated for 1 h at RT in 5% normal goat serum. Immunohistochemistry was performed by incubating the sections (overnight, at 4°C) with the following primary antibodies: Tomato Lectin (TL; 1:70, Sigma-Aldrich), and anti-Ki67 (1:500; ThermoScientific) diluted in PBST supplemented with 1% normal goat serum (NGS, Millipore). After washing, slices were incubated with the secondary antibody coupled to Alexa 633 (1:1,000, Invitrogen) in PBST for 2 h at RT.

### Image Processing and Data Analyses

The image processing was performed as previously described (García-Marqués and López-Mascaraque, 2013; Figueres-Oñate et al., 2016). Briefly, first the fluorescent labeling was visualized under an epifluorescence microscope (Nikon, Eclipse E600) with the appropriate filter cubes (Semrock): UV-2A (FF01-334/40-25), Cerulean (FF01-405/10), GFP (FF01-473/10), YFP (FF01-520/15), mKO (FF01-540/15), mCherry (FF01-590/20) and Cy5 (FF02-628/40-25). Images were acquired on a Leica TCS-SP5 confocal microscope, capturing the different XFPs in separate channels with the 20× objective. The wavelength of excitation (Ex) and emission (Em) for each XFP were (in nanometers, nm): mT-Sapphire (Ex: 405; Em: 520–535), mCerulean (Ex: 458; Em: 468–480), EGFP (Ex: 488; Em: 498–510), YFP (Ex: 514; Em: 525–535), mKO (Ex: 514; Em: 560–580), mCherry (Ex: 561; Em: 601–620), and Alexa 633 (Ex: 633; Em: 650–760). Confocal laser lines were in-between 25% and 40% in all cases and the maximum projection images were created using confocal (LASAF Leica) and NIH-ImageJ software. Cortical astroglial clones were defined as those cells sharing the same fluorescent marks, based on the fluorescence intensity and specific cellular location of the fluorophores (nucleus or cytoplasm). Those clones were located surrounding the perivascular infiltrates and the enlarged perivascular compartments, identified with TL expression. For the quantifications, we analyzed 10 clones/per animal in three EAE Star-Track electroporated mice. Statistical analysis of the data and graphical representations were performed using R statistical software version 3.5.0 (R Core Team, 2018) and Sigma Plot version 14.0 (Systat Software, San Jose, CA, USA).

## Results

### Identification of Cortical EAE Lesions

Although in the EAE MOG-induced model the main affectation is located in the spinal cord, EAE experiments in mice demonstrated that, as occurs in human MS, the cerebral cortex is also affected (Procaccini et al., 2015). In fact, neocortical lesions have previously been recognized to be present in MS (Stadelmann et al., 2008). In particular, chronic EAE induced by MOG peptide in mice showed, although with some variability, intracortical MS-like lesions characterized by diffused demyelination, astroglial and microglial activation, oligodendrocyte precursor cells (OPC) proliferation, and a low leukocyte/macrophage infiltrate (Girolamo et al., 2011; Mangiardi et al., 2011; Hasselmann et al., 2017).

**Figure 1:**
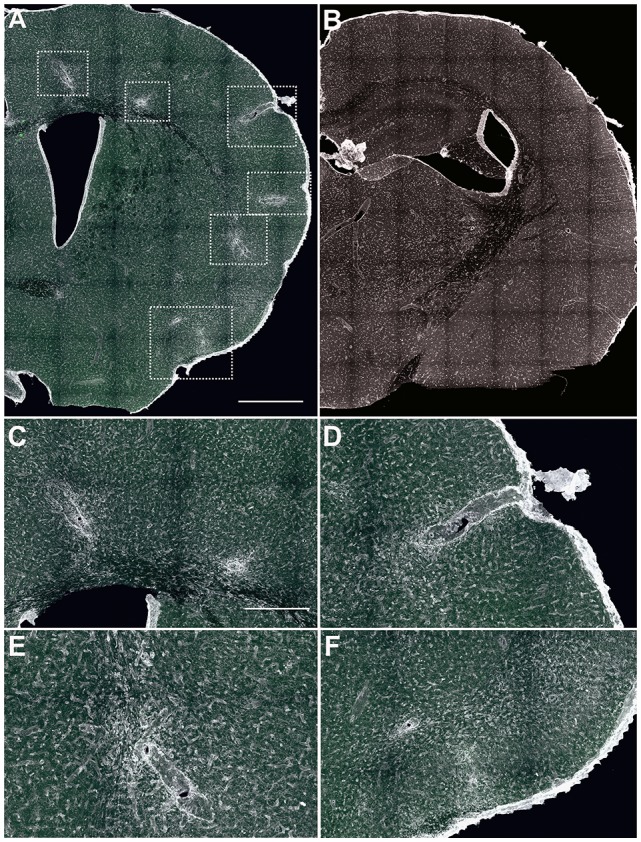
Identification of cortical lesions. **(A,B)** Low magnification images of brain coronal sections labeled with tomato lectin (TL) to label the infiltrates surrounding the lesions. In experimental autoimmune encephalomyelitis (EAE)-induced mice **(A)** cell infiltrates were located along the brain, while in sham animals **(B)**, no lesions or infiltrates were identified. In EAE animals, affected areas (boxed with dotted line) were subcortical white matter **(C,E)**, cortical/subcortical area **(C,D)** and layer I (subpial, **F**). Scale bars: **(A,B)** 400 μm and **(C–F)** 100 μm.

Results presented in Figure 1 correspond to animals previously StarTrack electroporated, both EAE (Figures 1A,C–F) and sham mice (Figure 1B). We initially performed Tomato-lectin immunohistochemistry, in both MOG-induced (Figures 1A,C–F) and sham animals (Figure 1B) to visualize the presence of inflammatory infiltrates, microglial cells and blood vessels. Affected regions were determined by the presence of perivascular inflammatory infiltrates and larger perivascular compartments located throughout the rostro-caudal axis of cortical gray and white matter (Figures 1A,C–F) of MOG-injected animals, similar to our previous results in wildtype mice (Matute et al., 2007; Zabala et al., 2018). No perivascular cuffs were found in sham-electroporated mice brains (Figure 1B).

**Figure 2:**
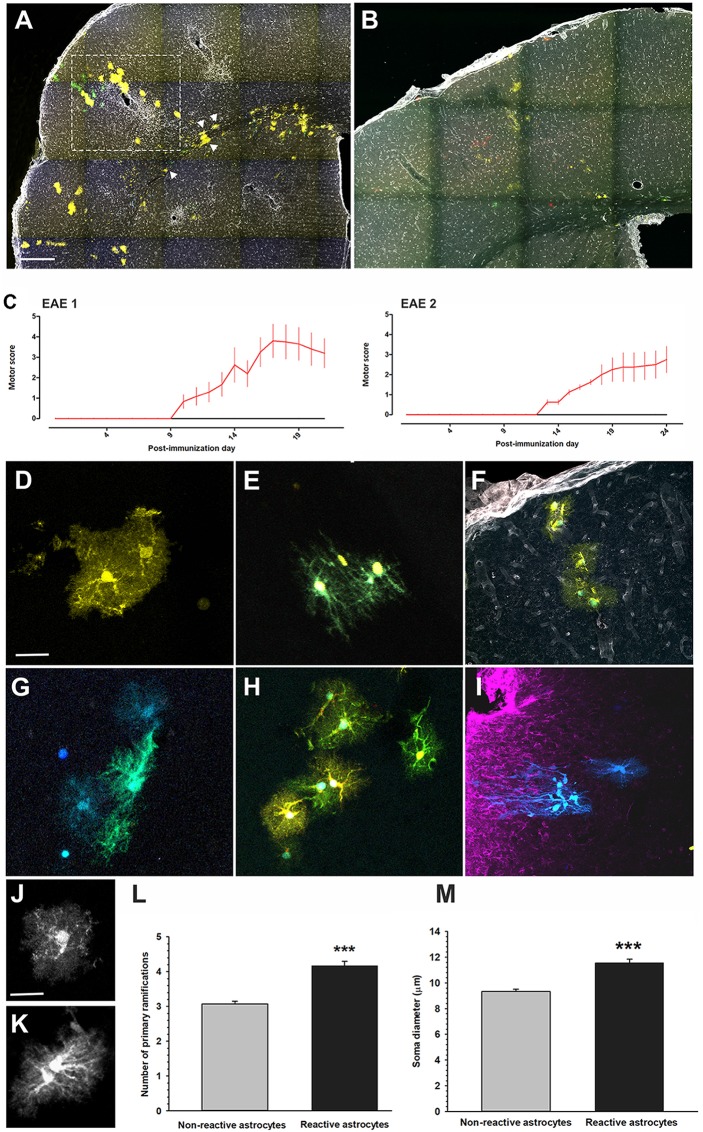
Cortical clonal dispersion in Star Track IUE-adult mice.** (A)** Low magnification image of a coronal cortical brain section of an EAE-induced adult mice *in utero* electroporated at E14. Analysis was restricted to the labeled sibling astrocytes surrounding the cortical lesion (dashed square). The injured area was determined by the presence of infiltrates close to the enlarge perivascular space (stained with TL, in white). Most clonal groups display protoplasmic morphologies throughout the cortical layers, but some clones of fibrous astrocytes were located in the subcortical white matter (arrowheads). **(B)** Low magnification image of a coronal cortical brain section of a sham adult mice *in utero* electroporated at E14. **(C)** Clinical score of both EAE groups (red) and controls (black) in StarTrack-electroporated mice. **(D)** StarTrack labeled protoplasmic astrocytes in a sham brain. **(E)** StarTrack labeled fibrous sibling astrocytes in a sham brain. **(F)** StarTrack labeled vascular sibling astrocytes in a sham brain. **(G,H)** Hypertrophic astrocytes in an EAE myelin oligodendrocyte glycoprotein (MOG)-injected electroporated mice. **(I)** Hypertrophied astrocytes around the wounded area of an StarTrack electroporated mice lesioned with the model of fine-needle cortical injury (Martín-López et al., 2013). **(J,K)** Image of a typical protoplasmic astrocyte (**J** of a hypertrophied astrocyte **K**). **(L,M)** Graphs show the quantification of the number of primary ramifications **(L)**, and soma diameter **(M)** of non-reactive astrocytes and reactive astrocytes in EAE electroporated mice (***P < 0.001). Scale bar: **(A,B)** 200 μm; **(D,G)** 20 μm; **(E)** 30 μm; **(F,I)** 50 μm; **(H)** 40 μm and **(J,K)** 25 μm.

We identified three types of cortical lesions: perivascular intracortical lesions, cortico/subcortical lesions (Figures 1A,C–E) and subpial lesions accompanied by meningeal inflammation (Figure 1F), which was more evident in those animals with higher clinical score. These data support that the inflammation of meninges is important in the induction and propagation of cortical pathology in MS (Lassmann, 2012). Even some infiltrates were located in the striatum (Figure 2A), we focused on the cerebral cortex since most StarTrack labeled cells were located in cortex.

### Astrocytes Display Different Morphologies Depending on the Localization of Perivascular Infiltration in EAE-Mice

*In utero* electroporated mice with the StarTrack mixture were divided in two groups: one EAE induced with MOG35−55 peptide at 8 weeks of age in complete Freund’s adjuvant on day 0, and a sham group (Figures 2A,B; see “Materials and Methods” section). Both clinical signs and score were monitored up to day 21 and 24, respectively, in each of the two EAE experiments (Figure 2C). Even there was some variability in the intensity of the symptoms among mice, the analysis of all tissues was done around the peak of the disease (Figure 2C). Mice began to show neurological deficits between days 10–13, reaching a maximum score (hindlimbs symptoms characterized by loss of muscle tone to hemiparalysis) around days 17–22 (Figure 2C). Although many types of cells reacted in EAE lesions, we focused on the analysis of StarTrack-labeled astrocytes to identify the astroglial clones. StarkTrack method allowed the analyses of the astrocyte clones heterogeneity, based on their morphology and localization.

To perform the clonal analysis, we selected cortical regions where the StarTrack labeled cells were located close to the EAE affected areas identified by the accumulation of perivascular inflammatory infiltrates and the presence of enlarge perivascular compartments (Figure 2A). In electroporated sham animals, the protoplasmic astrocytes located in the gray matter, displayed more primary processes and a higher degree of branching (Figures 2B,D,J) compared to fibrous astrocytes with small elongated cell bodies and long processes located in the white matter (Figure 2E). Further, vascular clones of astrocytes were also found surrounding blood vessels (Figure 2F) in the electroporated sham mice. In EAE–electroporated mice, astrocytes closer to the infiltrate evidenced the typical hypertrophic morphology (Figures 2A,G,H,K), characterized by an increment in the number of their cellular processes (Figure 2L) and by an enlargement of the cell soma (Figure 2M). Remarkably, EAE-reactive astrocytes (Figures 2A,G,H) appeared morphologically different than those we previously reported (Martín-López et al., 2013) after fine-needle cortical injury model (Figure 2I). In that model, reactive astrocytes displayed an asymmetric hypertrophy with thinner and longer processes pointing out to the injury core (Figure 2I), even though both models exhibited enlarged somas than in non-injured brains.

**Figure 3:**
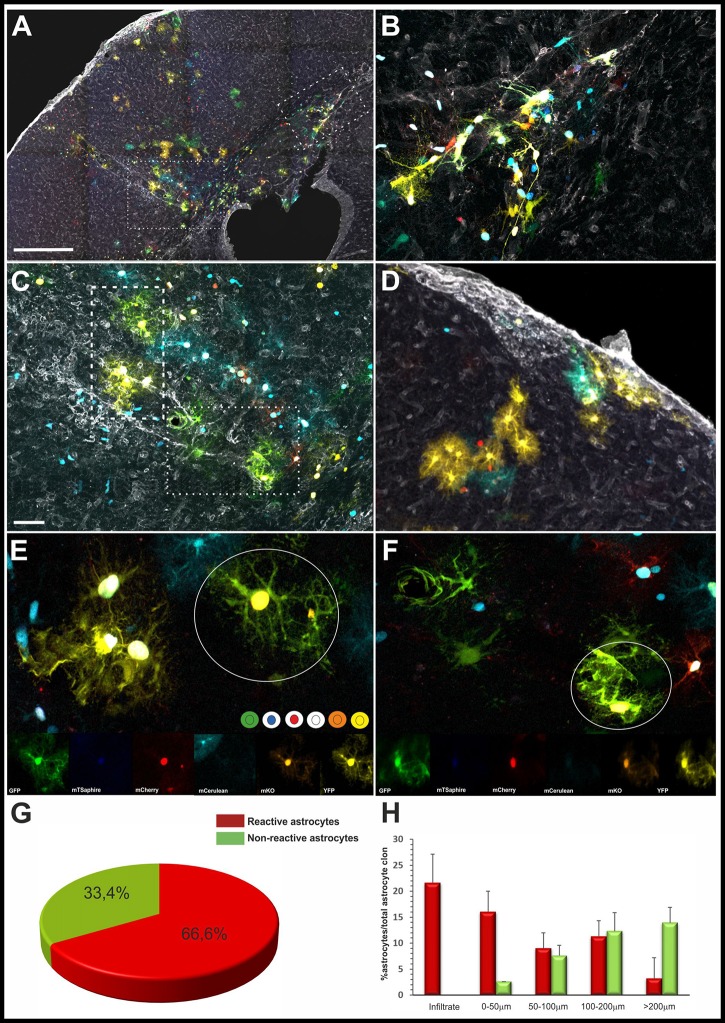
Astrocyte exhibit different reactive morphologies after EAE. **(A)** Low magnification of the clonal labeling in an EAE-StarTrack electroporated mouse. Perivascular inflammation in the subcortical white matter (**B**, dashed square), subcortical/intracortical areas (**C**, dotted square) and pia matter **(D)**. **(B)** Higher magnification of the boxed area in **(A)** shows hypertrophic fibrous astrocytes within the subcortical white matter. **(C)** Higher magnification of the dotted area in **(A)** shows protoplasmic astrocytes radially orientated embracing an enlarge perivascular compartment occupying several cortical layers. **(D)** A subpial infiltrate bordered by both pial and protoplasmic astrocytes clones. **(E,F)** Higher magnification of sibling reactive astrocytes with hypertrophic morphology showing thicker processes and soma. Qualitative clonal cell analysis based on the color combination and fluorophore cell location (nuclear or cytoplasmic) of clonally related astrocytes in the white circles. **(G)** The graph shows the quantification of the percentage of reactive and non-reactive astrocytes in Star-Track labeled clones. **(H)** Histogram showing the percentage of reactive and reactive astrocyte within the clones depending on their location with the lesion. Scale bar: **(A)** 400 μm; **(B,C)** 150 μm; **(D)** 80 μm; **(E,F)** 25 μm.

In all the cases, reactive astrocytes were identified by their characteristic reactive morphology (Figure 3). Reactive sibling astrocytes were analyzed in serial sections to decipher their morphology and precise clonal cell dispersion, independently of the lesion size. Astroglial clones located close to or within the subcortical white matter were exclusively composed of reactive fibrous astrocytes (Figures 3A,B), while those clones within the gray matter lesions were entirely composed of protoplasmic sibling astrocytes (Figures 3A,E,F). Astroglial clones related to subpial lesions were composed of a mix of both fibrous and protoplasmic astrocytes (Figure 3D). In addition, astrocytes with the same color code of fluorophores composition were assigned as sibling-cells defining the astrocyte clones (Figures 3E,F). The vast majority of the astroglial clones were composed by reactive astrocytes (Figure 3G). Further, the total number of hypertrophic and non-hypertrophic sibling astrocytes was quantified in relation to the distance from the inflammatory infiltrate (Figure 3H), showing a higher number of reactive astrocytes within 100 μm away from the inflammatory infiltrate.

### Clonal Analysis of Cortical Astrocytes in EAE-Induced Mice

**Figure 4:**
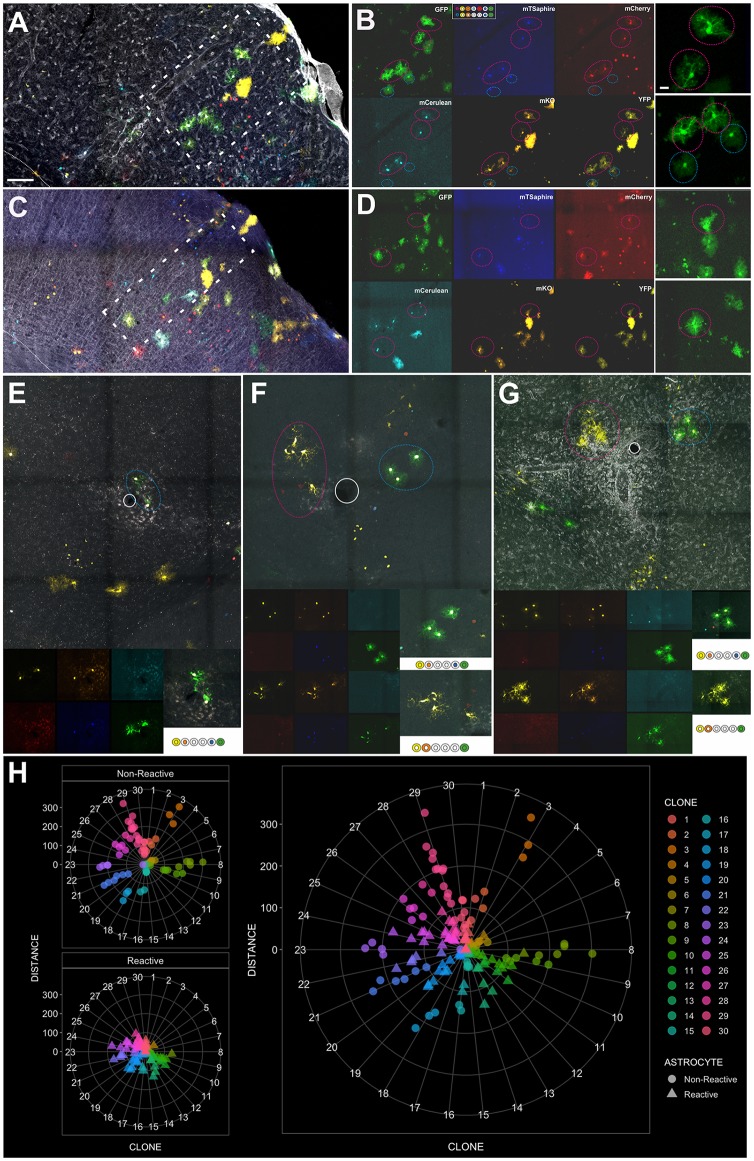
Clonally-related astrocytes targeted by IUE with StarTrack respond to EAE lesions. **(A,C)** Low magnification images of an EAE-electroporated mice. A protoplasmic astrocyte clone (white rectangle) surrounding a perivascular cuff in serial sections. **(B,D)** Qualitative cell analysis based on the color combination and fluorophore cell location (nuclear or cytoplasmic). Different clonal color-codes detailed by the expression of each fluorescent reporters: YFP, mKO, mCerulean, mCherry, mT-Sapphire and GFP. Sibling cells (pink circle), displaying a hypertrophic morphology and located close to the perivascular space. Sibling astrocytes located further (blue circle) displayed the typical protoplasmic morphology. **(E–G)** Clonally-related astrocytes with different morphologies depending on their location from the inflammatory infiltrate and their color-code. Scale bar: **(A,C)** 200 μm; **(B,D)**, inset **(E–G)** 20 μm and **(E–G)** 100 μm. **(H)** Radar plots showing the quantification of reactive and non-reactive astrocytes within 30 individual clones at different distances from the lesion.

To decipher how clonally-related cortical astrocytes respond in StarTrack electroporated EAE-induced mice, we performed a clonal analysis of cortical astrocytes surrounding the perivascular inflammatory areas. Astrocytes that were clonally-related displayed the same color-codes (Figures 3E,F, 4A–G).

Most clonally-related cortical astrocytes remained close to an inflammatory area and displayed hypertrophic morphologies, with enlarged cytoplasm and thick cellular processes and soma (Figures 4A–G). We analyzed individual clones in the EAE electroporated brains showing non-reactive astrocytes were mostly located far apart of the limit of the infiltrate (Figure 4H), while hypertrophic astrocytes were mainly located in the core of the infiltrate or really close to it (Figure 4H). The cumulative distribution of both populations, reactive and non-reactive astrocytes, is different according to the non-parametric Kolmogorov–Smirnov test (*D* = 0,528, *p* < 0.0001). In addition, it is worth to mention that 50% were composed exclusively by hypertrophic astrocytes, 20% by non-reactive astrocytes and 30% correspond to mixed clones (Figure 4H, reactive and non-reactive astrocytes in the same clone). Further, the presence of dividing cells was critically related to the lesion, we performed immunohistochemistry for the proliferation marker Ki67. However, not all StarTrack-labeled astrocyte in a specific area were positive for Ki67, just a low proportion of cells surrounding the lesions (33.4 ± 4.5%) was positive for this marker.

**Figure 5:**
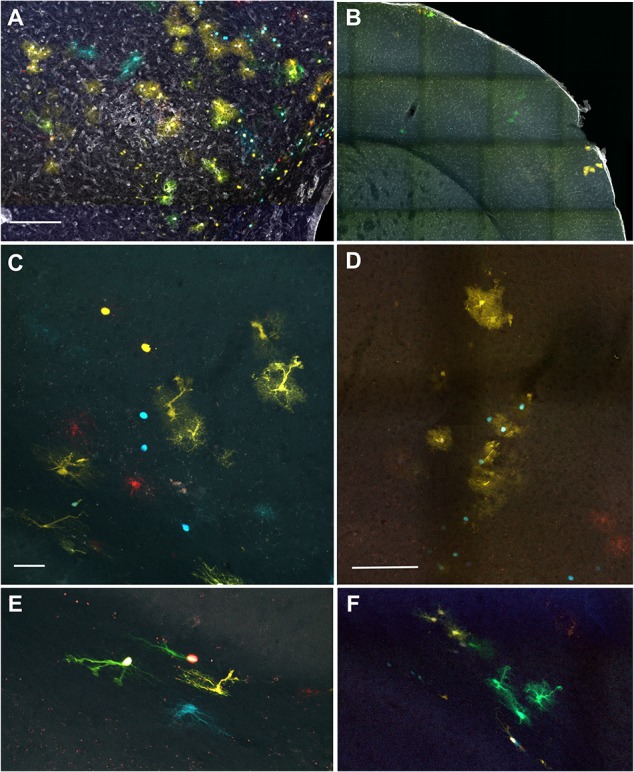
Hypertrophic clonally-related astrocytes are present out from the parenchymal inflammation infiltrates.** (A)** IUE-EAE StarTrack electroporated brain section stained with TL. **(B)** IUE-StarTrack sham electroporated brain section stained with TL. **(C)** Representative clonally-related cortical astrocytes with a reactive morphology arranged in a columnar manner in an IUE-EAE StarTrack electroporated mouse brain. **(D)** Representative clonally-related cortical astrocytes with the typical protoplasmic morphology in a sham StarTrack electroporated mouse. **(E)** Representative clonally-related fibrous astrocytes within a subcortical white matter in an IUE-EAE StarTrack electroporated mouse brain. **(F)** Representative clonally-related fibrous astrocytes in a sham StarTrack electroporated mouse. Scale bar: **(A)** 100 μm; **(B)** 200 μm; **(C,E)** 50 μm and **(D,F)** 100 μm.

Additionally, astrocyte clones with reactive morphologies (Figure 5) were located in areas without the presence of clearly identified inflammation or enlarged blood vessels (Figures 5A,C,E). Hypertrophied sibling cortical astrocytes (Figure 5C) were disposed in columnar disposition with different morphologies across the layers (Figure 5C). Within the subcortical white matter, clonally-related astrocytes displayed hypertrophied morphologies with enlarged soma and thicker processes (Figure 5E), On the contrary, in sham-StarTrack electroporated animals (Figures 5B,D,F) none hypertrophied astrocytes were found across the brain, neither in gray matter (Figure 5D) nor in subcortical white matter (Figure 5F). These data indicate that the inflammatory environment triggered in the EAE model also affects morphology and behavior of astrocytes in the cerebral cortex.

Since different type of astrocytes develop from specific progenitor cells, we expected that sibling astrocytes responded similarly to the brain damage. Nevertheless, these results revealed different clonal responses. Although the vast majority of the reactive clones acquired hypertrophic morphologies in relation to distance to the injury, others did not modify their morphologies, even at the same distance. We also provide evidence that not all the cells of a clone respond equally, but rather other factors, as distance or localization, are probably involved in their response.

## Discussion

Here, we report a heterogeneous behavior of sibling astrocytes in response to cortical damage induced by a demyelinating EAE-induced mice model. Whereas some cortical clones displayed all sibling astrocytes with reactive morphology, other clones, located at equivalent distances, did not display altered cell morphologies. In other cases only those astrocytes of a clone, closer to the core of the perivascular infiltration, showed hypertrophic cytoplasm. Additionally, most astroglial clones acquired hypertrophic morphologies in relation to the injury distance, suggesting a role of the environment in the response of the adjacent astrocytes. Thus, astroglial clones in the EAE model exhibited a varied response with some becoming hypertrophic, while other other clones located similar distances from the lesion were unresponsive as we previously described after a mechanical brain injury (Martín-López et al., 2013).

Astrocytes are the most abundant cell-type of the human brain playing a variety of roles in brain homeostasis and synaptic maturation (Sofroniew and Vinters, 2010; Pekny et al., 2016). After brain damage astrocytes suffer dramatic pathological changes, such as reactive gliosis and glial scar formation, including abnormal hypertrophy and massive proliferation of astrocytes. However, the molecular identity and cues that dictate the structural changes in reactive astrocytes remain unclear (Sofroniew and Vinters, 2010; Liddelow and Barres, 2017). Further, astrocytes must be considered another potential therapeutic target for MS, although few studies focused on the cortical gray matter (Ponath et al., 2017, 2018). However, no study to date addressed the clonal astrocytic response to these lesions.

In this study using MOG-induced EAE model, we showed that astrocytes displayed a reactive morphology displaying thicker cellular processes and soma (Brosnan and Raine, 2013; Correale and Farez, 2015; Eilam et al., 2018). Conversely, in this model hypertrophic astrocytes did not show the asymmetric morphology with cellular processes pointing the core of the lesion, as described after a mechanical brain injury (Martín-López et al., 2013). Likewise, in EAE-model the StarTrack labeled reactive astrocytes were located surrounding enlarged blood vessels and perivascular inflammatory cuffs, while other reactive astrocytes were located away from the perivascular inflammatory infiltrates. In fact, both types of injuries activate a cascade of cellular and molecular events aimed to restore the CNS homeostasis, minimizing the brain damage. While after traumatic injuries astrocytes are involved in the generation of the glial scar (Fawcett and Asher, 1999), in the EAE-induced model, astrocytes actively participate in both lesion development and repair (Brosnan and Raine, 2013; Correale and Farez, 2015; Ponath et al., 2018). In this regard, although reactive astrocytes share common properties, they also display unique cellular and molecular features that are specific to different neuropathologies (Pekny et al., 2016; Ponath et al., 2018). This large astrocyte heterogeneity, in response to injury, validates the importance to design selective strategies to promote CNS repair.

With the StarTrack methodology, which allows to target single embryonic progenitors, we provided the first *in vivo* evidence of the clonal response of astrocytes in a demyelinating scenario and their capacity to respond to an injury. Clonally-related astrocytes (sibling astrocytes) responded in a heterogeneous manner to the perivascular inflammatory cortical environment of EAE induced animals. Whereas in some clones all sibling cells exhibited the typical hypertrophic morphology of reactive astrocytes, other clones, located at similar distances, did not exhibit altered cell morphologies. In addition, several astrocyte clones with hypertrophic morphologies were located away from the perivascular/parenchymal inflammatory infiltrates. This suggest that EAE induction could generate a widespread neural inflammation that affects all the cortical astrocytes. These results resembled very closely those previously described in a traumatic brain injury model (Martín-López et al., 2013).

Further, astrocytes contribute to the innate immune response in MS, playing a critical role in both oligodendrocyte damage and axonal degeneration (Sofroniew and Vinters, 2010; Correale and Farez, 2015). In fact, reactive astrocytes are categorized as types A1 or A2 (in analogy to the “M1” and “M2” phenotype categories for macrophages) according to their transcriptome profiles and their role in inflammation/immune response (Liddelow and Barres, 2017). A1-type astrocytes, induced by inflammation, are abundant in MS and other neurodegenerative diseases, including Alzheimer/Parkinson diseases, whereas A2-type astrocytes, induced by ischemia, express neurotrophic factors (Liddelow and Barres, 2017; Liddelow et al., 2017). It is likely that, distinct astrocytic phenotypes concur during different phases of a pathological process: first, reactive astrocytes produce pro-inflammatory cytokines combined with hypertrophy and proliferation; and, in a second phase, they promote anti-inflammatory functions and neurodegeneration (Colombo and Farina, 2016). Actually, in MS lesions the reactive astrocytes drive inflammatory and neurotoxic responses, reduce inflammation and promote neuroprotection and lesion repair (Correale and Farez, 2015; Ponath et al., 2018). Furthermore, astrocytes contributed to the development of MS lesions and by extension to the progression of the disease, which highlights their critical role as early and active players in lesion pathology (Ponath et al., 2018). These data are in agreement with our results, even we just analyzed the astrocyte response in the peak score of the acute EAE phase, when probably just a subpopulation of astrocyte clones is reactive. Further clonal analyses at other stages of the disease (onset, remission) would reveal whether different lineages or cell clones are activated depending on the progression of the disease.

Our results also show that in some cases there is a distinct response of sibling cells to the inflammation. This could be related to the distance to the perivascular infiltrate or perivascular compartment, but it also could be due to the gradual expression of molecules within the lesion. There are many molecules and genes that are known to be expressed in MS lesions that affect the remyelination and also the progression of the lesion (Clemente et al., 2011; Piaton et al., 2011; Zeis et al., 2018). Several diffusible molecules could be implicated in this process, including basic fibroblast growth factor (bFGF), anosmin 1 or class 3 semaphorins (Clemente et al., 2011; Piaton et al., 2011). It is possible that, these molecules could affect those astrocytes located close to the lesion, in addition to other molecular cues secreted by both the inflammatory infiltrates and astrocytes (Legroux and Arbour, 2016).

The variability in the clonal response to brain damage clearly indicates that the heterogeneity of reactive astrocytes could be probably determined early in CNS development, but also by environmental cues present in the injured brain. Nevertheless, our findings showed a high heterogeneity in the astroglial response to inflammation that could be influenced by the signals received from the environment, their location respect to the affected brain area and factors during development. In fact, astroglial clonal response provides *in vivo* evidence of how brain development leaves a fingerprint of heterogeneity in astrocyte roles during the course of MS lesions. Further, these responses to brain injury could contribute to the inflammatory response, promoting lesion repair. Additionally, the presence of intrinsic factors, determined during the embryonic development and related to the cell lineage, could affect the astrocyte response. All these evidences should be taken into account for the further design of strategies and treatments for neurological diseases.

## Author Contributions

AB, LL-M and CM conceived and designed the experiments. AB performed electroporations and analyzed clonal data. FP-C and CM performed the EAE and tissue processing. AB and LL-M wrote the article, all authors revised and approved the manuscript.

## Conflict of Interest Statement

The authors declare that the research was conducted in the absence of any commercial or financial relationships that could be construed as a potential conflict of interest.
